# Repurposing Proteostasis-Modifying Drugs to Prevent or Treat Age-Related Dementia: A Systematic Review

**DOI:** 10.3389/fphys.2018.01520

**Published:** 2018-10-30

**Authors:** Daniel S. Heard, Camilla S. L. Tuttle, Nicola T. Lautenschlager, Andrea B. Maier

**Affiliations:** ^1^North West Mental Health, Melbourne Health, Melbourne, VIC, Australia; ^2^@AgeMelbourne, Department of Medicine and Aged Care, University of Melbourne, Melbourne, VIC, Australia; ^3^Academic Unit for Psychiatry of Old Age, Department of Psychiatry, University of Melbourne, Melbourne, VIC, Australia; ^4^@AgeAmsterdam, Department of Human Movement Sciences, Vrije Universiteit Amsterdam, Amsterdam Movement Sciences, Amsterdam, Netherlands

**Keywords:** aging, alzheimer's disease, dementia, lithium, proteostasis, rapamycin

## Abstract

**Background:** Dementia has a significant impact on quality of life of older individuals. Impaired proteostasis has been implicated as a potential cause of dementia, that can be therapeutically targeted to improve patient outcomes. This review aimed to collate all current evidence of the potential for targeting proteostasis with repurposed drugs as an intervention for age-related dementia and cognitive decline.

**Methods:** PubMed, Web of Science and Embase databases were searched from inception until 4th July 2017 for studies published in English. Interventional studies of repurposed proteostasis-modifying drugs in Alzheimer's disease (AD), Parkinson's disease (PD), Lewy Body disease, vascular dementia, and cognitive aging, in either animal models or humans with change in cognition as the outcome were included. The SYRCLE and Cochrane tools were used to assess risk of bias for included studies.

**Results:** Overall 47 trials, 38 animal and 9 human, were isolated for inclusion in this review. Drugs tested in animals and humans included lithium, rapamycin, rifampicin, and tyrosine kinase inhibitors. Drugs tested only in animals included Macrophage and Granulocyte-Macrophage Colony Stimulating Factors, methylene blue, dantrolene, geranylgeranylacetone, minocycline and phenylbutyric acid. Lithium (*n* = 10 animal, *n* = 6 human) and rapamycin (*n* = 12 animal, *n* = 1 human) were the most studied proteostasis modifying drugs influencing cognition. Nine of ten animal studies of lithium showed a statistically significant benefit in Alzheimer's models. Rapamycin demonstrated a significant benefit in models of vascular dementia, aging, and Alzheimer's, but may not be effective in treating established Alzheimer's pathology. Lithium and nilotinib had positive outcomes in human studies including Alzheimer's and Parkinson's patients respectively, while a human study of rifampicin in Alzheimer's failed to demonstrate benefit. Microdose lithium showed a strongly significant benefit in both animals and humans. While the risk of bias was relatively low in human studies, the risk of bias in animal studies was largely unclear.

**Conclusion:** Overall, the collective findings support the hypothesis that targeting proteostasis for treatment of dementia may be beneficial, and therefore future studies in humans with repurposed proteostasis modifying drugs are warranted. Larger human clinical trials focusing on safety, efficacy, tolerability, and reproducibility are required to translate these therapeutics into clinical practice.

## Introduction

Dementias including Alzheimer's disease, vascular dementia, Parkinson's disease and Lewy body disease, have a significant impact on global health due to the increasing number of older individuals suffering from this disease (Prince et al., 2015). Developing effective methods for preventing, delaying or treating dementia are pressing priorities. The highest risk factor for dementia is chronological age, with an annual incidence of Alzheimer's disease doubling every 5 years past the age of 65 years (Bermejo-Pareja et al., 2008). Dementia subtypes share several pathological processes including abnormal accumulation of misfolded proteins such as; amyloid beta (Aβ) and tau in Alzheimer's disease, and alpha-synuclein in Parkinson's disease and Lewy Body disease (Ganguly et al., 2017). Loss of proteostasis is an important feature during the aging process (López-Otín et al., 2013), suggesting the age-related decline in the ability to refold or degrade damaged proteins may contribute to the exponential rise in dementia incidence observed with increasing age (Yerbury et al., 2016).

Several drugs already approved for their use in humans are known to enhance proteostasis including; lithium, mTOR inhibitors (sirolimus/rapamycin, everolimus), and tyrosine kinase inhibitors (nilotinib). The concept of modifying aging with a repurposed drug to prevent multiple diseases of aging will soon be tested in the Targeting Aging with Metformin (TAME) trial. TAME will examine Metformin's ability to prevent diseases of aging in non-diabetic elderly, including cognitive impairment (Barzilai et al., 2016), via targeting the deregulated nutrient sensing associated with aging. Applying a similar strategy to target the loss of proteostasis could be effective in preventing and/or treating age-related dementia.

This systematic review will examine the evidence for targeting proteostasis with repurposed drugs as an intervention for age-related dementia and cognitive decline.

## Methods

### Protocol registration and search strategy

The protocol of this systematic review was registered at PROSPERO International prospective register of systematic reviews (Reg #: CRD42018091645). PubMed, Web of Science and Embase databases were used for this search from inception until 4th July 2017. The complete search strategy is presented in Supplementary Data 1. Key search terms included; “vascular dementia,” “Alzheimer^*^ disease” “Lewy Body Disease,” “Parkinson^*^ disease,” “cognitive aging,” “autophag^*^,” “lysosom^*^,” “proteasome endopeptidase complex,” “molecular chaperone^*^,” “unfolded protein response,” “insulin^*^,” “mTOR,” “GSK-3,” “akt,” “PI3K,” “AMPK,” “sirtuin^*^,” “sirolimus,” “everolimus,” “temsirolimus,” “rapamycin,” “metformin,” “DPP-4,” “GLP-1,” “nicotinamide,” “NAD,” “spermidine,” “imatinib,” “nilotinib,” “dasatinib,” “bosutinib,” “ponatinib,” “bafetinib,” “lithium,” “heat-shock protein,” “calori^*^ restriction,” “carbohydrate restricted diet,” “protein restricted diet”. In addition to the database search a “snowballing” method was used to identify relevant articles out of the reference section and PubMed citations of each included article. After duplicates were removed studies were then screened for inclusion using Covidence systematic review software *(Veritas Health Innovation, Melbourne, Australia)*.

### Eligibility criteria

#### Type of studies

The search was designed to retrieve all published research studies that investigated the effect of modifying protein homeostasis or deregulated nutrient sensing (DNS) on cognitive function in age-related neurodegenerative disease and normal aging populations. To be included in this review the study had to report on one or more neuropsychological tests measuring change in cognitive function. To meet the criteria of modifying protein homeostasis or deregulated nutrient sensing, the intervention had to be previously demonstrated to modulate these pathways, or data had to be provided proving the intervention's effect on these pathways. Animal *in vivo* models and human trials were included in this review. The following Dementia populations/models were specifically targeted; Alzheimer's disease, Vascular Dementia, Parkinson's disease and Lewy Body Disease. In addition, normal aging populations, defined as a population not suffering from dementia and over the age of 18 years for human studies, as well as populations likely to have a higher pace of aging such as animal models with diabetes or obesity were included. Randomized controlled trials (RCTs) and non-randomized studies comparing outcomes to either retrospective or prospective controls met the inclusion criteria. Studies were excluded if they met the following criteria; observational studies, exercise as the sole intervention, *in vitro* data only, conference abstracts, reviews, editorials, letters to the editor, case reports with ≤5 population size, or published in a language other than English.

#### Outcome

In animals (using mice as an example), cognitive tests would include spatial memory tests (Morris water maze [MWM], radial arm water maze [RAWM], Barnes maze), associative learning tasks (passive avoidance, fear conditioning), alternation tasks (Y-Maze/T-Maze), recognition memory tasks (Novel Object Recognition), attentional tasks (3 and 5 choice serial reaction time), set-shifting tasks, and reversal learning tasks. In human studies examples of neuropsychological measures would be cognitive testing batteries commonly used in clinical or research settings to examine cognitive function, such as the Mini-Mental State Examination (MMSE), Rowland Universal Dementia Assessment Scale (RUDAS), Neuropsychiatry Unit Cognitive Assessment Tool (NUCOG), Montreal Cognitive Assessment (MOCA), Clinical Dementia Rating Scale Sum of Boxes (CDR-SoB), Addenbrooke's Cognitive Examination (ACE) or Alzheimer's Disease Assessment Scale-cognitive subscale (ADAS-Cog).

### Study selection

Two review authors (DH and CT) independently screened the titles and abstracts and subsequently the full text articles of potentially relevant studies against the inclusion and exclusion criteria. A third reviewer (ABM) resolved any disagreements between the authors.

Included studies were separated into the following four groups for data extraction (1) proteostasis–repurposed drug, (2) proteostasis–novel intervention (defined as a novel molecule, botanical extract, or dietary manipulation), (3) DNS–repurposed drug and (4) DNS–novel intervention. Where an intervention is thought to modify both pathways (for example the mTOR inhibitor, rapamycin) it was included in the loss of proteostasis group. The current paper presents the results of the 1st group: proteostasis—repurposed drugs.

### Data extraction and quality assessment

The following variables were extracted independently by two reviewers (DH and CT): author, year of publication, study design, species, animal model/population (dementia subtype or normal aging), sample size, age, sex, baseline cognition/stage of disease, duration of study, cognitive outcome, drug, comparator, setting, hallmark(s) of aging targeted by the intervention, and journal citation. For binary outcomes the number of events and total number in group, percentage of events or ratios with confidence intervals; for continuous outcomes, mean or median, standard deviation, standard error, confidence intervals or interquartile range, and number of participants; other reported results such as mean difference and *p*-values of measures of cognitive function.

Risk of bias was assessed by two reviewers (DH, CT) using the Cochrane Risk of Bias tool (Higgins et al., 2011) for human studies and SYRCLE's risk of bias tool for animal studies. The SYRCLE RoB tool is an adaptation of the Cochrane tool for use in systematic reviews of laboratory animal studies (Hooijmans et al., 2014).

### Registered human trials

To establish the progress of repurposed drugs into human studies which have not yet been completed, clinicaltrials.gov was searched for registered studies of the drugs identified in our search in Alzheimer's disease, Parkinson's disease, Lewy Body Disease and Vascular Dementia.

## Results

### Study selection and characteristics

**Figure 1:**
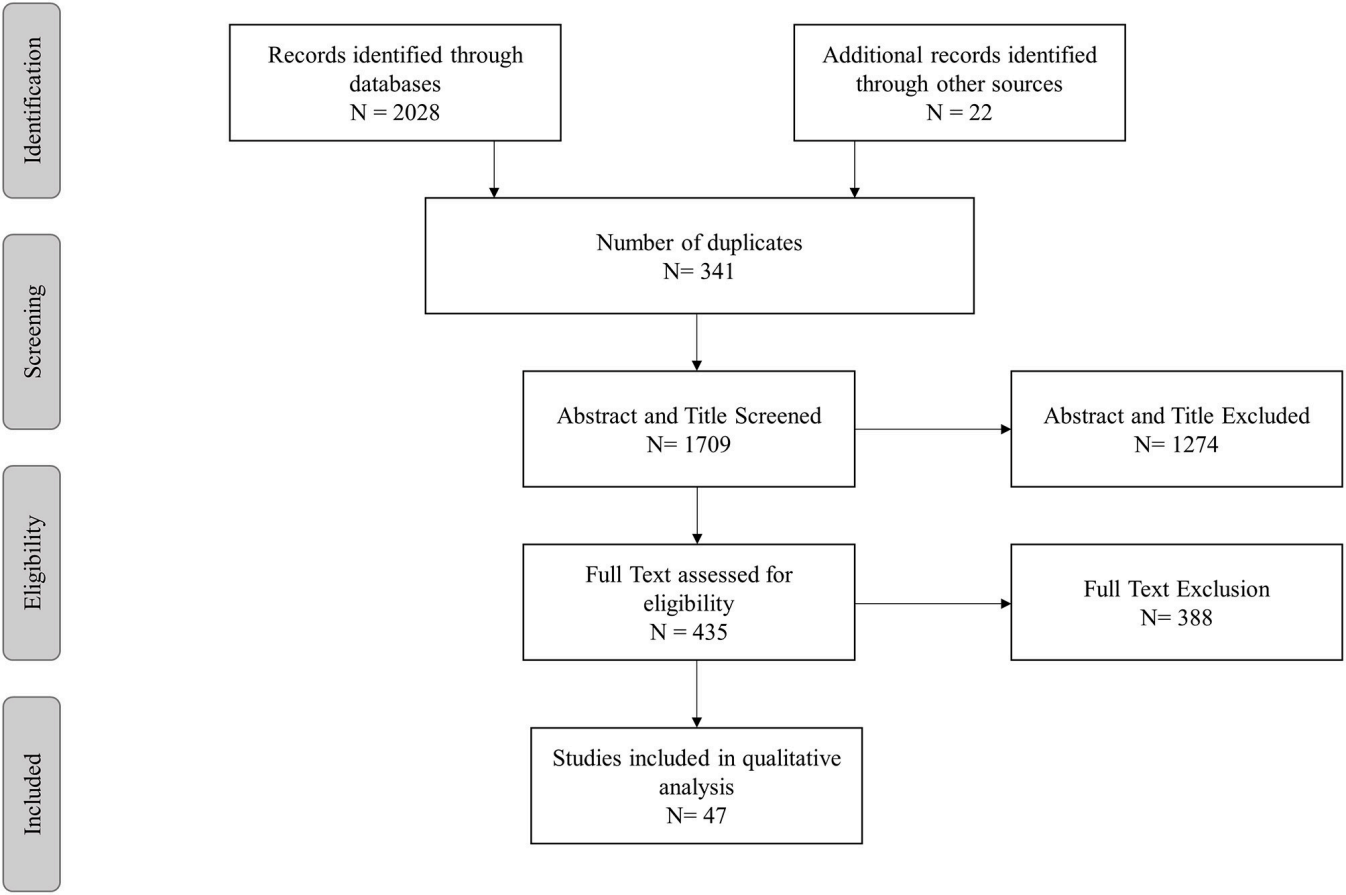
Study selection process.

**Figure 2:**
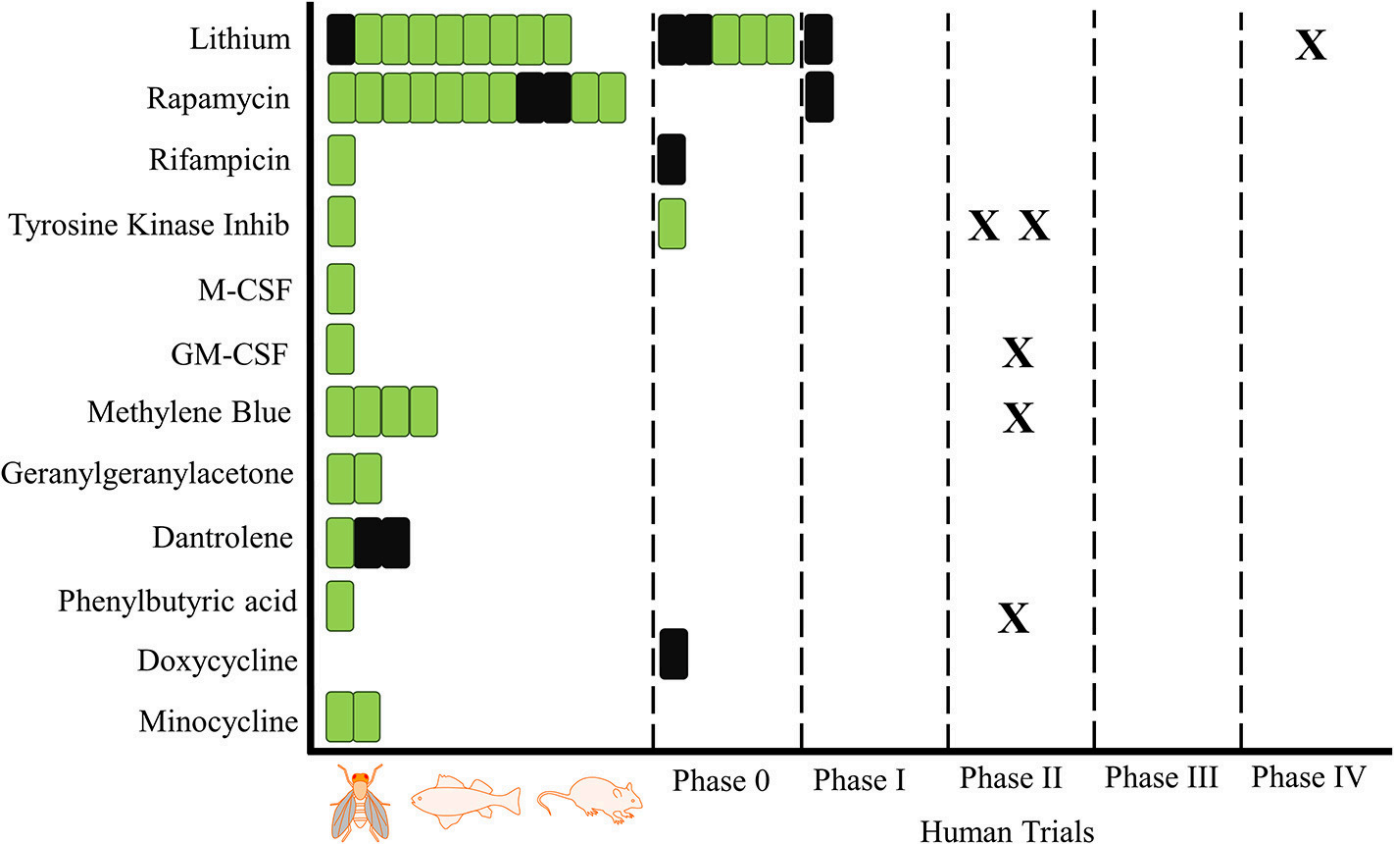
Proteostasis Drugs and Cognitive Outcomes: From Animals to Humans. A schematic overview of trials investigating the influence of proteostasis drugs on cognitive outcomes. One bar is equal to one trial, green indicates a positive result for at least one cognitive outcome and black is indicative of no positive outcomes. X represents a registered trial. Trials are arranged in chronological order.

The literature search and selection process for this review is illustrated in Figure 1. After exclusion of duplicates the remaining 1,687 studies were screened for Title and Abstracts of which 413 underwent full text screening. An additional 22 studies were identified via snowballing. Overall, 47 articles specifically investigating a proteostasis intervention on Dementia and cognitive aging were included in this review. The repurposed drugs used in these intervention studies are outlined in Tables 1, 2 and Supplementary Table 1. The following drugs were found testing the modification of cognition in animal and human studies; lithium (*n* = 10 animal, *n* = 6 human), rapamycin (*n* = 12 animal, *n* = 1 human), rifampicin (*n* = 1 animal, *n* = 1 human), tyrosine kinase inhibitors (bosutinib *n* = 1 animal, nilotinib *n* = 1 human), Macrophage Colony Stimulating Factor (M-CSF; *n* = 1 animal), Granulocyte Macrophage Colony Stimulating Factor (GM-CSF; n = 1 animal), methylene blue (*n* = 4, animal), geranylgeranylacetone (GGA; *n* = 2 animal), dantrolene (*n* = 3 animal), minocycline (*n* = 2 animal) and phenylbutyric acid (*n* = 1 animal). Doxycycline was tested in a single human trial only. Of these drugs only lithium, rapamycin, rifampicin, and the tyrosine kinase inhibitors have been tested in both animal and human studies (Figure 2).

**Table 1:** Characteristics of animal studies testing the effect of lithium (a), rapamycin (b), rifampicin (c), bosutinib (d) on cognition.

	**Author, Year**	**Species**	**Model**	**Sample size (*****n*****)**		**Age**	**Sex (%F)**	**Baseline cognition**	**Duration**	**Dose**
				**Rx**	**Ctrl**					
a	Caccamo et al., 2007	Mouse	AD (3xTg)	Wt: 10Tg: 10	Wt: 10Tg: 10	15m	NR	Est	4w	300μl of 0.6mol/L/d IP
a	Rockenstein et al., 2007	Mouse	AD (Tg hAPP)	WT: 6Tg: 6	WT: 6Tg: 6	3m	NR	Est	3m	20mg/kg/d IP
a	Fiorentini et al., 2010	Mouse	AD (TgCRND8)	Ear: 8Est: 8	Ear: 8Est: 8	2m6m	Mix	EarEst	5w	0.223mEq/L IP
a	Toledo and Inestrosa, 2010	Mouse	AD (Tg APP- PS1)	3-≥6	3-≥6	9m	NR	Est	12w	0.2–1.5 meq/L
a	Sy et al., 2011	Mouse	AD (3xTg)	Na = 6LPS = 6	Na = 6LPS = 6	11–13m	67	Est	6w	6–10mg/d food
a	Nunes et al., 2015	Mouse	AD (Cg- Tg(PDGFB- APPSwInd) 20Lms/2J)	Pre: 8Est: 7WT: NR.	WT: 12TG: 7	2m10m	0	PreEst	16m8m	0.25mg/kg/d (H2O)
a	Nocjar et al., 2007	Rat	Aging (Sprague-Dawley)	16	14	2m	0	Pre	80d	0.72mEq/l food
a	Wilson et al., 2017	Rat	AD (Tg McGill- R-Thy1-APP)	WT: ≥5Tg:≥5	WT: ≥5Tg:≥5	3m	Mix	Ear	2m	Li 40μg/kg/d PR
a	Nery et al., 2014	Zebrafish	AD (ICV Aβ)	No inj: 10Veh: 10 AB: 10	No inj: 10Veh: 10 AB: 10	5d	Mix	Pre	5d	100μm (H2O)
a	McBride et al., 2010	Drosophila	AD (Tg psn[B3]/+, psn[I2]/+) PD (Tg 30Y-GAL4:UAS-Syn)	Pre: 72	Pre: 70	30d	0	Pre	25d	5mM Li food
				Est: 74	Est: 75	45d		Est	15d	
				PD: 39	PD: NR					
									
b	Spilman et al., 2010	Mouse	AD (Tg hAPP) Aging (C57BL/6J)	12 10	12 10	7m	0	Ear YA	3m	14mg/kg food
b	Majumder et al., 2011	Mouse	AD (3xTg) Aging (C57BL6/ 129svj)	4040	20 20	18m	NR	PreEst	16m3m	14mg/kg food
b	Halloran et al., 2012	Mouse	Aging (C57BL/6J)	9–14	9–14	12m25m	Mix	MAOA	40w	14mg/kg food
b	Majumder et al., 2012	Mouse	Aging (C57BL/6/ 129svj)	2020	20	18m	NR	YAMA	16m3m	14mg/kg food
b	Lin et al., 2013	Mouse	AD (Tg hAPP)	Tg: 10	Tg: 10	7m	0	Est	16w	14mg/kg food
				WT: 18	WT: 17			YA	
b	Neff et al., 2013	Mouse	Aging (C57BL/6Jrj)	YA: 20	YA: 20	4m	0	YA	12m	14mg/kg food
				MA: 21	MA: 21	13m		MA		
				OA: 27	OA: 27	20–22m		OA		
b	Wang et al., 2014	Mouse	Aging (C57BL/6J, stz diabetic)	9	9	3m	0	Est	45d	2.24mg/kg/d PO
b	Lin et al., 2015	Mouse	AD (APOE4 Tg)	15	15	7m	100	Pre	6m	14mg/kg food
b	Jahrling et al., 2017	Mouse	VD (LDL-R–/– HFD)	10	10	12m	0	Est	16w	14mg/kg food
b	Zhang et al., 2017	Mouse	AD (3xTg)	10	10	7m	50	Ear	2m	1mg/kg/d PO
b	Wang et al., 2016	Rat - Sprague Dawley	AD (ICV Aβ)	18	20	6m	0	Pre	2w	500 microg ICV/2w
b	Zhu et al., 2014	Rat	AD (scop Wistar)	10	10	NR	0	Pre	14d	3.5mg/kg/d IP
c	Umeda et al., 2016	Mouse	AD (Tg APPOSK), (tau609) Aging (WT)	APPOSK 12m 0.5mg: 9 18m 0.5mg: 10 1mg: 10 Tau609 8m 0.5mg: 8 15m 1mg: 7	APPOSK 12m: 9 18m: 10 WT 8m: 10 12m: 10 15m: 11 18m: 16 Tau609 8m: 9 15m: 7	APP 11m 17m Tau 7m 14m	0	Est	1m	[5pt]0.5mg/d (APP12m, APP18m, tau8m) 1mg/d PO (APP18m, tau15m)
d	Lonskaya et al., 2013	Mouse	AD (ICV lentiviral Aβ42, C57BL6) AD (Tg APP model)	Aβ42: 12 Tg: 12	Aβ42: 12 Tg: 12	11m	NR	Est	3w	5mg/kg/d IP

*3xTg, triple transgenic; Aβ, amyloid beta; AD, Alzheimer's dementia; Ctrl, control; d, days; Ear, early disease; Est, established; F, female; hAPP, human amyloid precursor J20; HFD, High fat diet; ICV, intracerebroventricular; Inj, injection; IP, intra-peritoneal; LDL-R–/–, low density lipoprotein receptor knockout; Li, lithium; LPS, lipopolysaccharide; m, months; MA, middle age; NR, not reported; OA, Old Age; PD, Parkinson's disease dementia; PO, per oral; PR, per rectum; Pre, presymptomatic; Rapa, rapamycin; Scop, scopolamine; stz, streptozocin induced; Tg, transgenic; VD, vascular dementia; w, weeks; WT, wild type; YA, young adult*.

**Table 2:** Characteristics of human studies testing the effect of lithium (a), rapamycin (b), rifampicin & doxycycline (c) and nilotinib (d) on cognition.

	**Author, year**	**Design**	**Condition**	**Sample size (n)**		**Age (yrs)**		**Female (%)**	**Baseline cognition**	**Duration**	**Dose**
				**Rx**	**Ctrl**	**Rx**	**Ctrl**				
a	Pomara et al., 1983	OL(pre-post)	AD	7	NA	“Geriatric”		NR	NR	6w	0.53 mmol/L (mean at 6w)
a	Macdonald et al., 2008	OL (match ctrl)	AD	22	44	80.9 ± 7.9	81.2	59	MMSE 12–24	12m	0.3–0.8 mmol/L
a	Hampel et al., 2009	RCT	AD	33	38	68.2 ± 7.2	68.9 ± 8.3	52	MMSE 21–26	10w	0.5–0.8 mmol/l
a	Leyhe et al., 2009	RCT	AD	13	14	71.0 ± 9.0	69.4 ± 8.5	59	MMSE 21–26	10w	0.5–0.8 mmol/L
										
a	Forlenza et al., 2011	RCT	AD-MCI	23	22	70.9 ± 5.3	74.2 ± 6.5	NR	MCI	12m	0.25–0.5 mmol/L
										
a	Nunes et al., 2013	RCT	AD	58	55	77.0 ± 0.1	78.0 ± 0.76	66	MMSE 12–24	15m	300 μg/d
										
b	Kraig et al., 2018	RCT	Aging	11	14	80.4 ± 8.6	80.6 ± 7.9	28	OA	8w	1mg/d PO
										
c	Molloy et al., 2013	RCT	AD	Rif: 101 Dox: 102 Rif + dox: 101	102	Rif:78.6 (73.5–82.3) Dox: 78.7 (74.1–83.6) Rif+dox:79.2 (74.4–83.5)	78.6 (72.4–83)	50	MMSE 20–25	12m	Rif: 300mg/d Dox: 100mg BD Rif + dox: 300mg/d + 100mg BD
d	Pagan et al., 2016	OL (pre-post)	PD	12	NA	71.8 (49–89)	NA	25	MoCA 9–28	6m	Nilo 150mg or 300mg/d

*OL, open label; RCT, randomized controlled trial; AD, Alzheimer's Disease, PD, Parkinson's disease; LBD, Lewy Body Disease; MMSE, Mini mental state exam; MoCA, Montreal Cognitive Assessment; NR, not reported; SD, standard deviation; MCI, Mild Cognitive Impairment; Li, lithium; Ctrl, control; NA, Not applicable; D, days; W, weeks; M, months; Rx, treatment; Rif, rifampicin; Dox, doxycycline; PL, placebo; Nilo, nilotinib; Age, refers to age at baseline of study*.

### Lithium and cognitive aging

Lithium was the most investigated proteostasis modulator for cognitive aging. Overall 16 studies (6 humans, 6 mice, 2 rats, 1 drosophila, and 1 zebrafish) investigating the influence of lithium on cognitive aging were found. The majority of these studies utilized an Alzheimer's animal model or were conducted in an Alzheimer's population (Tables 1, 2). The findings of these studies were largely positive with all animal studies, bar one (Caccamo et al., 2007), reporting the use of lithium as having a statistically significant beneficial impact on at least one cognitive outcome irrespective of treatment duration (Table 3). The findings of the animal studies are consistent with the lithium human studies (Table 4), with three randomized controlled studies showing a statistically significant benefit of the use on lithium on either the ADAS-Cog or MMSE (Leyhe et al., 2009; Forlenza et al., 2011; Nunes et al., 2013). The other three studies, two of which were open-label (Pomara et al., 1983; Macdonald et al., 2008) and one RCT (Hampel et al., 2009), did not show cognitive benefits after treatment with lithium.

**Table 3:** Results of animal studies testing the effect of lithium (a), rapamycin (b), rifampicin (c) and bosutinib (d) on cognition.

	**Author, year**	**Cognitive tests**	**Outcomes**	**Significance**
a	Caccamo et al., 2007	T-maze (Alternation %)	Wt Li−66.67 (8.7), ctrl−72.15 (2.4)	±
			Tg Li−55.71 (5.6), ctrl−55.21 (5.8)	±
a	Nocjar et al., 2007	**Hole-board spatial discrimination task:**		
		Search time (s) session 6	Li−6 (1), Ctrl−20 (3)	+
		Repeat visits (# lower = better)	Li−0.6 (0.1), Ctrl−1.5 (0.4)	+
		Number of errors (# lower = better)	Li−1.6 (0.2), Ctrl−1.9 (0.2)	±
		**T-maze delayed alternation task:**		
		Sessions to reach criterion (#)	Li−13.5 (1), Ctrl−20 (1.5)	+
		**Social conditioned place preference:**		
		Percent correct (%)	1min Li−75 (2.5), Ctrl−72.5 (5)	±
			3min Li−70 (3), Ctrl−58 (4)	+
			5min Li−65 (2.5), Ctrl−60 (2.5)	±
		Run time (min)	1min Li−3.7 (1), Ctrl−1.9 (0.2)	NR
			3min Li−2.7 (0.5), Ctrl−2.8 (0.5)	±
			5min Li−2.9 (0.5), Ctrl−3.2 (0.5)	±
		Preference for social chamber (s):	Li−280 (100), Ctrl−175 (100)	+
a	Rockenstein et al., 2007	**Morris water maze:**		
		Meters to reach platform day 7	Tg Li−3 (0.5), ctrl−11.5 (3)	+
			Wt Li−3.25 (0.5), ctrl−3 (0.5)	
		Platform crosses (#)	Tg Li−7 (1.5), ctrl−6 (1)	±
			Wt Li−6 (2), ctrl−7 (1)	
		Time in target quadrant (s)	Tg Li−16 (3), ctrl−18 (2)	±
			Wt Li−15 (3), ctrl−16 (3)	
a	Fiorentini et al., 2010	**Morris water maze:**	Early stage disease (3 months)	
		Escape latency day 4 (s)	Li 35s (5s), ctrl 55s (2s)	+++
		Time in target section (%)	Li 7.4% (2.25%), ctrl 1.25% (1.25%)	++
		Inhibitory avoidance test (s)	Li 27 (3), ctrl 9.5 (2)	+++
			Late stage disease (7 months)	
		Escape latency day 4 (s)	Li 50 (2), ctrl 59 (1)	NR
		Time in target section (%)	Li 1.5 (0.5), ctrl 1.5 (0.5)	±
		Inhibitory avoidance test	Li 14 (3.5), ctrl 8 (4)	±
a	Toledo and Inestrosa, 2010	**Morris water maze:**		
		Escape latency day 5 (s)	Tg Li−45 (9), ctrl−35 (7.5)	±
			WT ctrl−27 (10)	
		**Memory flexibility test:**		
		No. of trials to criterion (#)	Tg Li−7 (0.5), ctrl 12 (0.25)	+
			WT ctrl−5 (0.25)	
a	Sy et al., 2011	**Morris water maze:**		
		Escape latency during day 7 (s)	Li + Na−25 (2), ctrl + Na−17 (5)	NR
			Li + LPS−26 (3), ctrl + LPS−20 (3)	NR
		**Probe trial (24 h)**		
		Time spent in target quadrant (s)	Li + Na−17.5 (4.5), ctrl + Na−22 (4)	±
			Li + LPS−19 (2), ctrl + LPS−13 (4)	±
		Latency to platform (s)	Li + Na−24 (6), ctrl + Na−17.5 (5)	±
			Li + LPS−26 (5), ctrl + LPS−47.5 (7.5)	+
		Number of platform location crosses (#)	Li + Na−5 (1.3), ctrl + Na−5.4 (1)	±
			Li + LPS−3 (0.25), ctrl + LPS−1 (0.5)	+
a	Nunes et al., 2015	**Barnes maze:**	Treated before deficits	
		Escape latency (s; mean)	Li−40 (3), ctrl−75 (7)	+
		Time in target quadrant (%)	Li−52.3 (6.8) Ctrl−22.8 (4.9)	+++
		Aversive memory test session (s)	Li−299 (298/300), Ctrl−216 (137/298)	++
		**Barnes maze:**	Treated after deficits	
		Escape latency (s; mean)	Li – 25 (2), Ctrl – 75 (7)	+
		Time in target quadrant (%)	Li−32 (4) Ctrl−22.8 (4.9)	++
		Aversive memory test session (s):	Li−298 (139/298), Ctrl−216 (137/298)	+
a	Wilson et al., 2017	**Novel object recognition (preference ratio)**	WT Li−0.39 (0.04), veh−0.43 (0.04) Tg AD Li−0.39 (0.02) veh−0.28 (0.02)	NR +
		**Morris water maze:**		
		Escape latency training day 5 (s)	WT Li−31 (7), veh−15 (5)	NR
			Tg Li−40 (10) AD veh−33 (8)	±
		Time in target quadrant (%)	WT Li−44 (5), veh−48 (5)	±
			Tg AD Li−45 (3), veh−50 (8)	±
		**Auditory fear conditioning task:**		
		Contextual (% freezing)	WT Li−60 (15), veh−79 (11)	±
			Tg AD Li−60 (10) veh−55 (10)	±
		Cued recall (% freezing)	WT Li−55 (15), veh−85 (10)	NR
			Tg AD Li−63 (7), veh−30 (5)	+
a	Nery et al., 2014	**Avoidance behavior**		
		% animals in non-stimulus area	Aβ inj Li 65 (2), ctrl: 55 (1)	+++
a	McBride et al., 2010	*Treated before deficits*	Alzheimer's Tg:	
		**Learning during training (%)**		
		psn[B3]/+ flies	Li 75(5) -> 45(10), ctrl 62.5(7.5) -> 51 (9)	+++
		psn[I2]/+ flies	Li 65(7.5) -> 30(10) ctrl 63(8) -> 52(8)	+++
		**Short term memory (%)**		
		psn[B3]/+ flies	Li–Naive 90(2), trained 70(5)	++
			Ctrl–Naive 76(6), trained 75(6)	±
		psn[I2]/+ flies	Li–Naive 88(2), trained 72(5)	+
			Ctrl–Naive 83(5), trained 85 (3)	±
		*Treated after deficits*		
		**Learning during training (%)**		
		psn[B3]/+ flies	Li 65(7.5) -> 18(7), ctrl 70(5) -> 62.5(7.5)	+++
		psn[I2]/+ flies	Li 76(5) -> 18(7), ctrl 47.5(7.5) -> 35(7.5)	+++
		**Short term memory (%)**		
		psn[B3]/+ flies	Li–Naive 90(4), trained 62.5(7.5)	++
			Ctrl–Naive 70(8), trained 75(7)	±
		psn[I2]/+ flies	Li–Naive 84(6), trained 62.5(7.5)	++
			Ctrl–Naive 57.5(7.5), trained 63(8)	±
		*Treated before deficits*		
		**Short term memory**	Parkinson's Tg:	
			Li 80(4) -> 75(5), ctrl 82.5(5) -> 78(4)	±
b	Spilman et al., 2010	**Morris water maze:**		
		Escape latency day 4 (s)	Tg Rapa 32(3), ctrl 42(5)	+
			WT Rapa 15(2.5), ctrl 32.5(3)	NR
		Platform crosses (#)	Tg Rapa 2.5(0.5), ctrl 0.9(0.1)	+/±
			WT Rapa 5(1), ctrl 3.1(0.4)	NR
b	Majumder et al., 2011	**Morris water maze:**		
		Escape latency day 5 (s)	Pre-AD–Rapa 26.9(2.1), ctrl 37.96(2.9)	+
			Est AD – Rapa 36(2), ctrl 37.96(2.9)	±
			YA–Rapa 20.7(1.05), ctrl 29.1(2.7)	+
			MA–Rapa 32.5(1.5), ctrl 29.1(2.7)	±
		Trial time in target quadrant (s)	Pre-AD–Rapa 22.5(2.5), ctrl 15(2.5)	+
			Est AD–Rapa 17.5(1.5), ctrl 15(2.5)	±
			YA–Rapa 29(2), ctrl 21.5(1.5)	+
			MA–Rapa 21(1.5), ctrl 21.5(1.5)	±
		MWM platform crosses (#)	Pre-AD–Rapa 3.5(0.5), ctrl 1.95(0.25)	+
			Est AD–Rapa 1.75(0.2), ctrl 1.95(0.25)	±
			YA–Rapa 5.25(0.3), ctrl 3.8 (0.25)	+
			MA–Rapa 3.5 (0.2), ctrl 3.8 (0.25)	±
		Novel object recognition	Pre-AD–Rapa 65 (7), ctrl 50 (5)	+
			Est AD–Rapa 55 (2.5), ctrl 50 (5)	±
			YA–Rapa 67.5(2.5), ctrl 70 (4)	±
			MA–Rapa 60 (5), ctrl 70 (4)	±
b	Halloran et al., 2012	**Passive avoidance test (s)**	MA–Rapa 200(40), ctrl 160(40)	±
			OA–Rapa 200(30), ctrl 100(20)	+
b	Majumder et al., 2012	**Morris water maze:**		
		Escape latency day 5 (s)	YA–Rapa 21(1), ctrl 30(2.5)	+
			MA–Rapa 31(2), ctrl 30(2.5)	±
		Time in target quadrant (s)	YA–Rapa 28.73(1.65), ctrl 21.3(1.24)	++
			MA–Rapa 20.97(1.18), ctrl 21.3(1.24)	±
		Latency to platform (s)	YA–Rapa 20(2), ctrl 27(3)	+
			MA–Rapa 31(3), ctrl 27(3)	±
		Platform crosses	YA–Rapa 5.3(0.2), ctrl 3.9(0.15)	+++
			MA–Rapa 3.5(0.25), ctrl 3.9 (0.15)	±
b	Lin et al., 2013	**Morris water maze:**		
		Escape latency day 5 training (s)	WT–rapa 28(4), ctrl 25(3)	
			AD–rapa 35(9), ctrl 40(4)	±
		Platform crosses (#)	WT rapa−3.1(0.5), ctrl 3.9(0.6)	
			AD rapa−2.1 (0.5), ctrl−0.75 (0.25)	+
b	Neff et al., 2013	**Object place recognition (s)**	YA rapa–novel 22(3), known 12(2)	±
			YA veh–novel 24(4), known 14(2)	
			MA rapa–novel 15(3), known 10(2)	±
			MA veh–novel 17(2), known 15(4)	
		**Morris water maze:**		
		Escape latency day 5 (s)	YA rapa 30(1), veh 41 (2)	+/±
			MA rapa 37 (3), veh 39 (1)	+/±
		Time in target quadrant (s)	YA rapa 25(2), veh 21 (2)	++
			MA rapa 25 (2), veh 20 (3)	++
		Target crossings (#)	YA rapa 2.3(0.2), veh 1.3(0.2)	++
			MA rapa 1.5 (0.3), veh 1.5 (0.2)	±
		**Context fear conditioning:**		
		Activity suppression (ratio)	YA rapa 0.2 (0.02), veh 0.21 (0.02)	++
			MA rapa 0.195 (0.01), veh 0.28 (0.02)	++
			OA rapa 0.195 (0.01), veh 0.25 (0.04)	++
b	Wang et al., 2014	**Morris water maze:**		
		Escape latency day 4 (s)	Rapa 25(3), ctrl 35(4)	+
		Escape latency trial (s)	Rapa 16(5), ctrl 32(2.5)	++
		Time in target quadrant (s)	Rapa 26(3.5), ctrl 12.5(1)	++
b	Zhu et al., 2014	**Morris water maze:**		
		Escape latency (s)	Scop + rapa−50 (7.5)	−
			Scop + saline−38 (5)	++
			Saline only−55 (7)	
			Scop + rapa + MAD−39 (4)	+
		Time in target quadrant (%)	Scop + rapa−65 (7)	−
			Scop + saline−80 (8)	+
			Saline only−62 (6)	
			Scop + rapa + MAD−75 (7.5)	+
b	Lin et al., 2015	**Morris water maze:**		
		Escape latency (s)	Rapa 25(1), ctrl 19(2)	±
		Platform crosses (#)	Rapa 1.6(0.25), ctrl 1.75(0.25)	±
b	Wang et al., 2016	**Y-maze (alternation %):**		
		4wks post infusion	Rapa 39(6), ctrl 62(10)	−
		8 wks post infusion	Rapa 48(7), ctrl 53(8)	±
b	Jahrling et al., 2017	**Morris water maze:**		
		Escape latency day 4 (s)	Rapa 30(4), ctrl 40(5)	+++
		Trial time in target quadrant (%)	Rapa 32(6), ctrl 13(2)	+
		Spatial Novelty (>0.33 = intact)	Rapa 0.44 (0.02), ctrl 0.34 (0.02)	+++
b	Zhang et al., 2017	**Morris water maze:**		
		Escape latency day 5 (s)	Rapa−32.5(5), veh 67(13)	+++
		Time in target quadrant (%)	Rapa−42.5(7.5), veh 22.5(7.5)	+
		Number of platform crossings (#)	Rapa−3.75 (5.5), veh−1.5 (0.5)	+
c	Umeda et al., 2016	**Morris water maze:**		
		Escape latency day 5 (s)	12m APP rif−19(5), veh−35(6)	++
			18m APP veh−36(5)	
			18m APP rif0.5mg−29(5)	±
			18m APP rif1mg−17.5(5)	++
			8m Tau609 rif0.5mg 14(2.3), veh−41(8)	+
			15m Tau609 rif1mg 29(7), veh 43(7)	±
		Time in target quadrant (%)	12m APP rif−45(5), veh−29(3)	±
			18m APP veh−29(6)	
			18m APP rif0.5mg−37(6)	±
			18m APP rif1mg−49(4)	+
			8m Tau609 rif0.5mg 30(4), veh−16(7)	+
			15m Tau609 rif0.5mg 42(10), veh−21(9)	±
d	Lonskaya et al., 2013	**Morris water maze:**		
		Time in target quadrant (%)	Aβ icv bosu 29(1), ctrl 19 (1)	+
		Time in target quadrant (% of WT)	Tg bosu 87.5(15) ctrl 75(10)	+
		Platform crosses (#)	Aβ icv bosu 5.5 (0.5), ctrl 4 (0.25)	+
		Platform crosses (% WT)	Tg bosu 147.5(7.5), ctrl 80(5)	+

*+++ favoring intervention, highly significant p < 0.001. ++ favoring intervention, significant p < 0.01. + favoring intervention, significant p < 0.05. +/± trend favoring intervention, p < 0.1. ± not significant. +/± trend favoring control, p < 0.1. –favoring control, significant p < 0.05. – favoring control, significant p < 0.01. — favoring control, highly significant p < 0.001. LPS, lipopolysaccharide; MA, treated from middle age; MAD, 3-methyladenine; NR, p-value not reported; OA, treated from old age; Scop, scopolamine; YA, treated from young adulthood*.

**Table 4:** Results of human studies testing the effect of lithium (a), rapamycin (b), rifampicin & doxycycline (c) and nilotinib (d) on cognition.

	**Author, year**	**Cognitive tests**	**Outcome**	**Significance**
a	Pomara et al., 1983	Buschke selective reminding test	[2pt]No quantitative data reported—“None of the psychometric measures showed either consistent, significant increases or decreases”	±
		Digit span/supraspan test		
		Sperling test of iconic memory		
		Word fluency tasks		
		Wechsler Memory Scale		
a	Macdonald et al., 2008	Change in MMSE	Li−4.8 (5.5), ctrl−4.0 (5.0)	±
			
a	Hampel et al., 2009	MMSE	Li−23.6 (1.6) -> 22.6 (3.5)	±
			PBO−23.6 (1.7) -> 23.2 (2.7)	
		ADAS-Cog	Li−15.8 (4.2) -> 15.6 (4.4)	±
			PBO−5.4 (5)-> 16.6 (5.1)	
		ADAS-Cog % with improvement >4 points	Li−28.6%, PBO−14.3%	NR
			
a	Leyhe et al., 2009	ADAS-Cog	Li 19.2 (5.7) -> 17.7 (5.8)	+
			PBO 16.5 (5.1) -> 18.0 (5.1)	
a	Forlenza et al., 2011	ADAS-Cog	Li 11.0(6.7)->12.6(6.6), PBO 10.7(5.1)-> 13.9(8.5)	+
		CDR–SoB	Li 1.4(1.3) -> 2.2(1.8), PBO 1.9(1.4) -> 2.8(2.3)	±
		Delayed recall	Li 4.8(2.1) -> 4.8(2.2), PBO 4.2(2.3) -> 4.5(2.3)	±
		Figure recall	Li 2.3(1.2) -> 2.0(1.3), PBO 1.9(1.1) -> 1.6(1.2)	±
		Sequence letters & numbers	Li 6.4(2.1) -> 6.0(2.9), PBO 6.3(2.6) -> 5.1(2.6)	+
		Trail making test A (s)	Li 69.1(44.2) -> 62.8(31.5), PBO 89.9(67.4) -> 63.6(41.9)	±
		Trail making test B	Li 171.8(83.9) -> 184.9(78.1), PBO 207.1 (79.6) -> 190.7 (92.8)	±
		Conversion MCI->AD	Li (*n* = 20) Stable = 16, Progress = 4	±
			PBO (*n* = 20) Stable = 13, Progress = 7	
		MCI->AD converters CDR-SoB	Li 3.3(1.3) -> 4.4(1.5), PBO 3.4(1.4) -> 5.6(1.5)	+
a	Nunes et al., 2013	MMSE	Li 19.48 (0.67) -> 19.82 (0.9)	+++
			PBO 17.95 (0.73) -> 14 (1.326)	
b	Kraig et al., 2018	Pre-post test change		
		EXIT25	PBO 0.38 (-1.84, 2.61), rapa−0.1 (-3.31, 3.11)	±
		SLUMS	PBO 0.38 (-2.03, 1.26), rapa−0.8 (-3.92, 2.32)	±
		TAPS	PBO−1 (-3.18, 1.18), rapa 1.44 (-1.68, 4.57)	±
c	Molloy et al., 2013	SADAS-Cog	Rif−0m = 22, 12m = 27.5	—
			Doxy – 0m = 21, 12m = 25.5	—
			Rif + Doxy−0m = 22, 12m = 28	—
			PBO−0m = 21, 12m = 25	
		CDR-SoB mean	Rif−0m = 6, 12m = 8.5	±
			Non-Rif−0m = 5.75, 12m = 7.75	
			Doxy – 0m = 6, 12m = 8.5	±
			Non-Doxy – 0m = 5.75, 12m = 7.8	
		SMMSE	ns vs. placebo, data NR	±
		Qmci	Rif worse than PBO, data NR	—
d	Pagan et al., 2016	MMSE (change 0w->24w)	150mg–+3.85, 300mg–+3.5	NR
		SCOPA-Cog (change 0w->24w)	150mg–+1.85, 300mg–+2.00	

*+++ favoring intervention, highly significant p < 0.001. ++ favoring intervention, significant p < 0.01. + favoring intervention, significant p < 0.05. +/± trend favoring intervention, p < 0.1. ± not significant. +/± trend favoring control, p < 0.1. - favoring control, significant p < 0.05. – favoring control, significant p < 0.01. — favoring control, highly significant p < 0.001. EXIT25, Executive interview; NR, p-value not reported; PBO, placebo; RoB, Cochrane risk of bias; SLUMS, St Louis University Memory Status; TAPS, Texas Assessment of Processing Speed*.

### Rapamycin and cognitive aging

Rapamycin has been identified as the second most frequently investigated (*n* = 13 studies) proteostasis modulator. Twelve animal studies (10 mice, 2 rats), predominately using an Alzheimer's disease model, investigated the influence of rapamycin on cognitive aging (Table 1). Overall, nine out of twelve animal studies reported a statistically significant benefit on at least one cognitive outcome, irrespective of treatment duration (Table 3). One human study investigating safety, efficacy and tolerability of rapamycin in humans (Kraig et al., 2018), did not report any significant benefit to overall cognition in an older population (Table 4).

### Rifampicin, tetracycline antibiotics, tyrosine kinase inhibitors and cognitive aging

Rifampicin, tetracycline antibiotics and tyrosine kinase inhibitors have been tested in both animal and human models (Tables 1, 2). The two rifampicin studies (1 mouse, 1 human; Tables 3, 4) investigating its therapeutic effects for Alzheimer disease had opposite findings, with positive cognitive outcomes in mice (Umeda et al., 2016), but no benefit found in the human study (Molloy et al., 2013).

Studies of tetracycline antibiotics showed similar results to rifampicin, with minocycline showing cognitive benefits in two studies using rat and chicken models of Alzheimer disease (Supplementary Table 1, Supplementary Table 2), while another tetracycline, doxycycline, showed no benefit in human AD patients, either alone or in combination with rifampicin (Molloy et al., 2013).

The tyrosine kinase inhibitor, bosutinib, was reported in mouse models of Alzheimer's disease as statistically beneficial to cognitive function, and another tyrosine kinase inhibitor, nilotinib, was found to improve scores on the MMSE and SCOPA-Cog in an open-label study in patients with Parkinson's disease, however the statistical significance was not reported (Tables 3, 4).

### Other proteostasis-modifying drugs

There were six proteostasis modulators that have been tested to improve cognitive outcomes in animal models but are yet to be studied in human populations—M-CSF, GM-CSF, methylene blue, GGA, dantrolene, and phenylbutyric acid (Supplementary Table 1). Both of the M/GM-CSF studies indicated beneficial outcomes with the use of these therapeutics in mouse models of Alzheimer's disease, as was the case with methylene blue, GGA and phenylbutyric acid (Supplementary Table 2). Studies of dantrolene to improve cognitive outcomes showed a statistically significant improvement in one mouse model of Alzheimer's (Peng et al., 2012) but no benefit to cognition in another (Wu et al., 2015), with one study in aged rats indicating a trend toward benefit on Morris water maze performance.

### Risk of bias

Table 5 shows the SYRCLE risk of bias ratings for animal studies. The majority of animal studies had an unclear risk of bias, as specific details of randomization and blinding were often not provided. Most studies provided information on the baseline characteristics of animals, and some studies did specify that the investigator performing behavioral assessments of the animals was blinded to the treatment status of the animal, indicating low risk of bias where this was the case. Overall the risk of bias was similar across studies regardless of the drug being tested.

**Table 5:** SYRCLE Risk of Bias for animal studies.

**Author, year**	**Sequence generation**	**Baseline characteristics**	**Allocation concealment**	**Random Housing**	**Blinding of personnel & participants**	**Random outcome assessment**	**Incomplete outcome data**	**Selective outcome reporting**
**LITHIUM**								
Caccamo et al., 2007	Unclear	Low	Unclear	Unclear	Unclear	Unclear	Low	Low
Nocjar et al., 2007	Unclear	Low	Unclear	Unclear	Low	Unclear	Low	Low
Rockenstein et al., 2007	Unclear	Unclear	Unclear	Unclear	Unclear	Unclear	Low	Low
Fiorentini et al., 2010	Unclear	Low	Unclear	Unclear	Unclear	Unclear	Low	Low
Toledo and Inestrosa, 2010	Unclear	Unclear	Unclear	Unclear	Unclear	Unclear	Low	Low
Sy et al., 2011	Unclear	Low	Unclear	Unclear	Unclear	Unclear	Low	High
Nunes et al., 2015	Unclear	Low	Unclear	Unclear	Unclear	Unclear	Low	Low
Wilson et al., 2017	Unclear	Unclear	Unclear	Unclear	Unclear	Unclear	Low	Low
Nery et al., 2014	Unclear	Low	Unclear	Unclear	Unclear	Low	Low	Low
McBride et al., 2010	Unclear	Low	Unclear	Unclear	Low	Unclear	Low	Low
**RAPAMYCIN**								
Spilman et al., 2010	Unclear	Low	Unclear	Unclear	Unclear	Unclear	Low	Low
Majumder et al., 2011	Unclear	Unclear	Unclear	Unclear	Unclear	Unclear	Low	Low
Halloran et al., 2012	Unclear	Unclear	Unclear	Unclear	Unclear	Unclear	Low	Low
Majumder et al., 2012	Unclear	Unclear	Unclear	Unclear	Unclear	Unclear	Low	Low
Lin et al., 2013	Unclear	Low	Unclear	Unclear	Low	Unclear	Low	Low
Neff et al., 2013	Unclear	Low	Unclear	Unclear	Low	Unclear	Low	Low
Wang et al., 2014	Unclear	Low	Unclear	Unclear	Unclear	Unclear	Low	Low
Zhu et al., 2014	Low	Low	Unclear	Unclear	Unclear	Unclear	Low	Low
Lin et al., 2015	Unclear	Low	Unclear	Unclear	Low	Unclear	Low	Low
Wang et al., 2016	Unclear	Low	Unclear	Unclear	Unclear	Unclear	Low	Low
Jahrling et al., 2017	Unclear	Low	Unclear	Unclear	Low	Unclear	Low	Low
Zhang et al., 2017	Unclear	Low	Unclear	Unclear	Unclear	Unclear	Low	Low
**RIFAMPICIN**								
Umeda et al., 2016	Unclear	Low	High	Unclear	High	Unclear	Low	Low
**BOSUTINIB**								
Lonskaya et al., 2013	Unclear	Low	Unclear	Unclear	Low	Unclear	Low	High
**M-CSF**								
Boissonneault et al., 2009	Unclear	Low	Unclear	Unclear	Low	Unclear	High	High
**GM-CSF**								
Boyd et al., 2010	Unclear	Unclear	Unclear	Unclear	Unclear	Unclear	Low	High
**METHYLENE bLUE**								
Deiana et al., 2009	Low	Unclear	Unclear	Unclear	Unclear	Unclear	Low	Low
Hochgräfe et al., 2015	Unclear	Low	Unclear	Unclear	Unclear	Unclear	Low	Low
Medina et al., 2011	Unclear	Low	Unclear	Unclear	Unclear	Unclear	Low	High
Stack et al., 2014	Low	Low	Unclear	Unclear	Unclear	Unclear	Low	Low
**GGA**								
Hoshino et al., 2013	Unclear	Unclear	Unclear	Unclear	Unclear	Unclear	Low	Low
Sun et al., 2017	Unclear	Unclear	Unclear	Unclear	Unclear	Unclear	Low	Low
**DANTROLENE**								
Hopp et al., 2014	Unclear	Low	Unclear	Unclear	Unclear	Unclear	Low	Low
Peng et al., 2012	Unclear	Low	Unclear	Unclear	Unclear	Unclear	Low	Low
Wu et al., 2015	Unclear	Low	Unclear	Unclear	Unclear	Unclear	Low	Low
**PHENYLBUTYRIC ACID**								
Wiley et al., 2011	Unclear	Low	Unclear	Unclear	Low	Unclear	Low	Low
**MINOCYCLINE**								
Choi et al., 2007	Unclear	Low	Unclear	Unclear	Unclear	Unclear	Low	High
Gibbs and Gibbs, 2013	Unclear	Low	Unclear	Unclear	Unclear	Unclear	Low	Low

Table 6 shows the Cochrane risk of bias rating for human studies. There was significant heterogeneity among lithium studies, with (Pomara et al., 1983; Macdonald et al., 2008) scoring high risk of bias across most or all domains due to an open label design and not reporting all quantitative outcome data. (Hampel et al., 2009) had an intermediate risk of bias as investigators were aware of patient treatment status. Studies by Leyhe et al. (2009), Forlenza et al. (2011) and Nunes et al. (2013) scored a lower risk of bias due to randomized, double-blind designs, though details of sequence generation and allocation concealment were not reported. Studies of rapamycin (Kraig et al., 2018) and rifampicin (Molloy et al., 2013) scored a low risk of bias across most domains. The study using nilotinib (Pagan et al., 2016) was rated as having a high risk of bias across most domains, due to an open label design.

**Table 6:** Cochrane Risk of Bias for human studies.

**Author, year**	**Sequence generation**	**Allocation concealment**	**Blinding of personnel & participants**	**Blinding of outcome assessment**	**Incomplete outcome data**	**Selective outcome reporting**
**LITHIUM**						
Pomara et al., 1983	High	High	High	High	High	Unclear
Macdonald et al., 2008	High	High	High	High	High	High
Hampel et al., 2009	Low	Unclear	Low	High	Low	Low
Leyhe et al., 2009	Unclear	Unclear	Low	Unclear	Low	Low
Forlenza et al., 2011	Unclear	Unclear	Low	Low	Low	Low
Nunes et al., 2013	Unclear	Unclear	Low	Low	Low	Low
**RAPAMYCIN**						
Kraig et al., 2018	Low	Unclear	Low	Low	Low	Low
**RIFAMPICIN**						
Molloy et al., 2013	Low	Low	Low	Low	Low	Low
**NILOTINIB**						
Pagan et al., 2016	High	High	High	High	Low	Low

### Registered human trials

Figure 2 shows the progress of the drugs identified in our search from animal into human studies, including planned or ongoing studies registered on clinicaltrials.gov. Nilotinib has two phase 2 studies registered, one in Alzheimer's disease and one in Parkinson's disease. Lithium has one phase 4 study registered, while GM-CSF and phenylbutyric acid each have one phase 2 study registered in Alzheimer's disease.

## Discussion

In this review we have summarized all current animal and human research studies that have investigated the effect of proteostasis modulators on cognitive function. Overall, the therapeutic alteration of proteostasis pathways using repurposed drugs is a promising approach to the treatment of dementia and age-related cognitive decline with a reasonable research translation between animal and human studies showing similar conclusions observed across the studies.

### Lithium

Lithium was the most studied proteostasis modifying drug identified in this review, and the furthest progressed in translation to treatment of age-related dementia. All except one of the animal studies included in this review showed a benefit to at least one cognitive outcome despite heterogeneity in species, model, dose, stage of disease at intervention and duration of treatment. Three of six human studies also found benefit, and consistent with the results in mice the largest effect was observed with microdoses and long duration of treatment (Nunes et al., 2013). The only negative RCT used a high dose for a short duration (Hampel et al., 2009). Taken together, these studies show lithium has a consistent benefit in Alzheimer's disease in animal models and humans, which appears more pronounced with lower doses, longer durations of treatment, and when commenced at an earlier stage of disease.

The apparent superiority of lower doses of lithium is encouraging, as at standard psychiatric dose it can have renal and thyroid-related side effects that limit tolerability and is toxic at levels only slightly above the therapeutic range (Timmer and Sands, 1999). Higher lifetime exposure to natural microlevels of lithium in drinking water is associated with a reduced incidence of dementia (Kessing et al., 2017), a finding which adds plausibility to the idea that microdoses of lithium rather than the current therapeutic doses might be beneficial for the treatment of dementia.

A possible explanation for lithium's non-linear dose response involves the interaction between lithium's autophagy-enhancing inhibition of inositol triphosphate receptor (IP3R) signaling, and its autophagy-reducing inhibition of GSK-3B, a tau-phosphorylating kinase (Sarkar et al., 2008). Genetic reduction of IP3R signaling in drosophila rescues Alzheimer's disease phenotypes in the same way lithium does (McBride et al., 2010), suggesting lithium's inhibition of IP3R signaling via reducing the formation of inositol triphosphate (IP3) may be sufficient to cause its benefits. This is mechanistically plausible, as inhibition of IP3R signaling enhances autophagy through decreasing calcium release from the endoplasmic reticulum, enhancing proteostasis (Sarkar et al., 2005). However; lithium's inhibition of GSK3B has also been postulated to have beneficial effects in Alzheimer's disease via reducing tau phosphorylation and neurofibrillary tangle formation as well as inhibiting autophagy via increasing mTOR signaling (Sarkar et al., 2008). Although, this latter theory is less likely to explain the positive influence of microdoses of lithium as lower levels of lithium do not inhibit GSK-3B in mice (Nunes et al., 2015). However, this GSK-3B mechanism may explain the reduced efficacy of lithium observed with increasing doses. Increased mTOR signaling due to GSK3B inhibition counteracts the autophagy-enhancing effects of reduced IP3R activation beyond a certain dose (Sarkar et al., 2009). If increased mTOR activity limits the effective dose of lithium, this raises the question of whether an mTOR inhibitor such as rapamycin combined with lithium would have beneficial synergistic effects in dementia or cognitive aging. A synergistic benefit of lithium and rapamycin has been demonstrated in a drosophila Huntington's disease model (Sarkar et al., 2008), but to our knowledge this has not been explored in models of Alzheimer's disease.

### Rapamycin

Predictably, rapamycin the known inhibitor of mTOR was also one of the most studied proteostasis modulators investigated, more so in animal models then in humans. Overall, the trials indicated a positive therapeutic effect of rapamycin on cognitive outcomes. Cognitive benefits via rapamycin was demonstrated in models of normal aging (Majumder et al., 2011, 2012; Neff et al., 2013; Wang et al., 2014), vascular dementia (Jahrling et al., 2017), and transgenic Alzheimer's disease models (Spilman et al., 2010; Majumder et al., 2011; Lin et al., 2013; Zhang et al., 2017), which suggests rapamycin has potential to rescue cognitive decline caused by a range of pathologies. This breadth of efficacy is a promising characteristic, as autopsy studies have demonstrated that mixed pathology is common in dementia sufferers (Nelson et al., 2016).

Majumder et al. (2011) reported a possible mechanism explaining their findings that rapamycin while effective in preventing cognitive decline before pathology develops, fails as a treatment once Alzheimer's pathology is established. They found rapamycin induces autophagy strongly both before and after Alzheimer's pathology is present. However, increased autophagy induction fails to reduce levels of amyloidβ in mice with established disease and leads to accumulation of enlarged autophagosomes containing undigested material. This finding suggests deficient substrate clearance and is consistent with previous findings that autophagy in Alzheimer's disease is principally defective at the stage of autolysosomal proteolysis (Nixon and Yang, 2011; Bordi et al., 2016). Therefore, to be effective in established Alzheimer's dementia rapamycin may need to be combined with a drug that can enhance autophagy at the stage of autolysosomal digestion.

Rapamycin's demonstrated ability to improve phenotypes of aging in animals has recently led to human trials assessing safety and efficacy in older adults (Kraig et al., 2018). Despite demonstrating low-dose rapamycin can be used safely in older adults, they were unable to show significant enhancement of cognition. However, larger trials are required to determine the potential benefit of rapamycin's for cognitive aging in humans.

### Rifampicin, tetracycline antibiotics and tyrosine kinase inhibitors

Rifampicin was more effective when started earlier in the disease process, and when used at a higher dose (Umeda et al., 2016). Cohorts treated with the lower dose at a later stage of disease did not show an improved cognitive benefit, a finding which is relevant to interpreting the DARAD study of rifampicin in Alzheimer's disease by Molloy et al. (2013).

In the DARAD study, rifampicin was tested in Alzheimer's patients at a low dose and showed either no benefit or on some measures a significant worsening of cognition compared to placebo. A possible explanation for this failure is an insufficient dose and treatment duration. Data supporting this view is provided by Iizuka et al. (2017), who in an observational study determined that a minimum dose rifampicin of 450 mg/day for at least 12 months was required before any cognitive improvement was observed (Iizuka et al., 2017).

Doxycycline also failed to produce benefit for human Alzheimer's disease patients in the DARAD study (Molloy et al., 2013), despite positive animal studies with closely related tetracycline antibiotic minocycline (Choi et al., 2007; Gibbs and Gibbs, 2013). It is difficult to know what implications this has for minocycline's repurposing potential, however currently there are no upcoming human studies registered on clinicaltrials.gov for either doxycycline or minocycline in dementia.

Lonskaya et al examined bosutinib in two mouse models of established Alzheimer's disease, and demonstrated statistically significant benefits after 3 weeks of treatment. Beneficial effects on cognition were also observed in human Parkinson's disease patients by Pagan et al. although the small size and open label design of the study mean the results require confirmation in larger randomized trials. These promising findings have led to significant interest in repurposing these drugs, to the extent phase 2 clinical trials are currently assessing the effect of these drugs in Alzheimer's and Parkinson's disease cohorts.

### Other proteostasis-modifying drugs

Three other proteostasis-modifying drugs have excited interest in translating promising animal study findings into humans—GM-CSF, methylene blue and phenylbutyric acid have registered phase 2 studies on clinicaltrials.gov to test their use in Alzheimer's disease.

Dantrolene is a drug of interest due to similarities it shares with lithium, in that it enhances autophagy by reducing calcium efflux from the endoplasmic reticulum (Wang et al., 2017). Unlike lithium however it acts by inhibition of the ryanodine receptor rather than IP3R signaling, raising the possibility of a complementary mechanism of action (Vervliet et al., 2017). This suggests dantrolene is worth testing in human trials to determine whether it can provide similar benefits to lithium, and in combination with lithium in animal models for potential synergistic effects. Currently however, no human trials are registered on clinicaltrials.gov for dantrolene in dementia.

## Limitations

Our study has several limitations. Our search strategy was based primarily on key terms related to the mechanisms of proteostasis, with the addition of a selection of drugs well known to modulate these processes. Therefore, our search may have missed studies that examine proteostasis-modifying drugs not named in our search and not mentioning proteostasis related key terms. However, we addressed this by adding relevant articles by snowballing.

Secondly, because the present study's focus is restricted to approved drugs, it does not provide an adequate overview of the translational pipeline where a repurposed drug is used as the basis for novel molecules that proceed into later stage studies.

Third, we cannot exclude publication bias, particularly in animal studies which are unlikely to be registered beforehand and may be less likely to be published if results are negative.

## Conclusions

The results of this review support the concept of a translational approach to repurposing proteostasis modifying drugs for the treatment of age-related dementia and cognitive decline. However, larger clinical trials assessing the influence of these drugs particularly, lithium and rapamycin are required before they are ready for the clinic. In addition, animal models assessing whether the combination of proteostasis modulators can act in synergy to improve cognitive outcomes are required. A translational strategy based on systematic screening of rational drug combinations starting in simple model organisms such as C. elegans may provide a pipeline of novel candidate therapies to advance into human studies.

## Author contributions

DH: Search strategy, screening, data extraction, drafting manuscript; CT: Search strategy screening, data extraction, drafting manuscript; NL: Search strategy, drafting manuscript; AM: Search strategy, conflict resolution, drafting manuscript.

### Conflict of interest statement

The authors declare that the research was conducted in the absence of any commercial or financial relationships that could be construed as a potential conflict of interest.
