# Supplementary material for: Repurposing Proteostasis-Modifying Drugs to Prevent or Treat Age-Related Dementia: A Systematic Review
Source: Front Physiol. 2018 Oct 30;9:1520. doi: 10.3389/fphys.2018.01520 (PMC6218672; doi:10.3389/fphys.2018.01520)
Supplement: Supplementary file 1 [file Table_1.pdf]

Supplementary Material

Repurposing proteostasis-modifying drugs to prevent or treat age-related dementia: a systematic review.

Daniel S. Heard[1], Camilla S.L. Tuttle[2], Nicola T. Lautenschlager[1,3], Andrea B. Maier [2,4*]

[1]North West Mental Health, Melbourne Health, Melbourne, Victoria, Australia

[2]@AgeMelbourne, Department of Medicine and Aged Care, University of Melbourne, Melbourne, Victoria, Australia.

[3]Academic Unit for Psychiatry of Old Age, Department of Psychiatry, University of Melbourne, Melbourne, Victoria, Australia.

[4.] @AgeAmsterdam, Department of Human Movement Sciences, Vrije Universiteit Amsterdam, Amsterdam Movement Sciences, The Netherlands.

*** Correspondence:**
Prof Andrea. Maier
andrea.maier@unimelb.edu.au

Supplementary Data 1 – Search strategy

The search query was as follows:

(Vascular dementia OR Alzheimer* Disease OR Lewy Body Disease OR Parkinson* Disease OR Cognitive Aging)

AND

(autophag* OR lysosom* OR Ubiquitin* OR Proteasome endopeptidase complex OR molecular chaperone* OR unfolded protein response OR insulin* OR mTOR OR GSK-3 OR akt OR PI3K OR AMPK OR sirtuin* OR sirolimus OR everolimus OR temsirolimus OR rapamycin OR metformin OR DPP-4 OR GLP-1 OR nicotinamide OR NAD OR spermidine OR imatinib OR nilotinib OR dasatinib OR bosutinib OR ponatinib OR bafetinib OR lithium OR heat-shock protein OR calori* restriction OR carbohydrate restricted diet OR protein restricted diet)

AND

cognition

Supplementary Table 1 – Characteristics of animal studies testing the effect of M-CSF (e), GM-CSF (f), Methylene blue (g), Geranylgeranylacetone (h), Dantrolene (i), Phenylbutyric acid (j) and Minocycline (k) on cognition.

	Author, year	Species	Animal Model	Sample size		Baseline age	Female (%)	Baseline severity	Duration	Dose
				Rx	Ctrl					
e	Boisson-neault 2009	Mouse	Tg AD (APPSwe/ PS1)	13<br>13	15<br>12	6m<br>9m	0	Pre<br>Est<br>Est	4m<br>3m	40 mg/kg IP
f	Boyd 2010	Mouse	Tg AD (AβPPsw/ PS1)	WT: 9<br>Tg: 5	WT: 8<br>Tg: 6	12m	Mix	Est	20d	5μg/d subcut
g	Deiana 2008	Mouse	AD (scop induced)	AD MB<br>0.15: 10<br>0.25: 10<br>0.5: 10<br>1:12<br>4:11<br>MB/Riva: 14<br>WT MB<br>4:11<br>MB/Riva: 9	AD: 12<br>WT: 13	2-3m	100	Est	95min	mg/kg IP: 0.15, 0.25, 0.5, 1, 4. MB 0.15 + riva 0.1 (inj 5m post scop)
g	Hochgrafe 2015	Mouse	AD (Tg TauΔK and TauRDΔK)	All 6-11:<br>TauΔK<br>1.5m<br>9m<br>15m<br>TauRDΔK<br>1.5m<br>12m<br>15m	WT:<br>6-11	1.5m<br>9m<br>15m	NR	Pre<br>Ear<br>Est	14.5m<br>6m<br>3m	20mg/kg/d PO or 40mg/kg/d (TauRDΔK 12m group only)
g	Medina 2011	Mouse	AD (3xTg)	WT: 13<br>3xTg: 17	WT: 10<br>3xTg: 14	6m	0	Est	16w	25mg/ 100g chow
g	Stack 2014	Mouse	AD (Tg P301L)	WTlow: 14<br>Wthigh: 15<br>Tglow: 14<br>Tghigh: 12	WT: 10<br>Tg: 9	1m	Mix	Pre	9m	4mg/kg chow (low)<br>40mg/kg chow (high)
h	Hoshino 2013	Mouse	AD (Tg APP23)	15	15	3m	100	Ear	9m	1.25g/kg bwt/d chow

h	Sun 2017	Mouse	AD (Tg APP/PS1)	Tg200: 8<br>Tg400: 8<br>Tg800: 8<br>WT800: 8	WT: 8<br>Tg: 8	3m	0	Pre	9m	200/400/ 800mg/d chow
i	Hopp 2014	Rat	Aging<br>F-344 rats	6<br>12	6<br>11	3m<br>22m	0	YA<br>OA	4w	5mg/kg/d SC
i	Peng 2012	Mouse	AD (3xTg)	3xTg: 5	WT: 12<br>3xTg: 10<br>3xTg Veh: 5	2m	NR	Pre	11m	icv inf 25mM/28 d for 90d, then 5mg/kg SC 3x weekly for 8m.
i	Wu 2015	Mouse	AD (3xTg)<br>Aging (C57BL/6)	3xTg: 14<br>WT: 14	3xTg: 16<br>WT: 12	16m	Mix	Pre*	6m	5mg 2x weekly PO gavage
j	Wiley 2011	Mouse	AD (Tg APP/PS1) Aging (C57BL/ 6J)	Wt: 9<br>Tg: 10	WT: 10<br>Tg: 13	2m	0	Pre	14m	1mg/g^{-1}/d in water
k	Choi 2007	Rat	AD (ICV Aβ)	Sham: 8<br>AD: 10	Sham: 11<br>AD: 8	7w	0	Est	4w	45mg/kg/day IP for 3w
k	Gibbs 2013	Chicken	AD (ICV Aβ)	All n=12-16<br>10min pre<br>5min pre<br>0min pre inj<br>5min post	AD:12-16	1d	0	Pre Est	6h	300 pmol/hemi-sphere ICV

*3xTg mice normally show deficits by 16m, but no difference between 3xTg and WT ctrls found at baseline by these investigators.

3xTg=triple transgenic. AD = Alzheimer's dementia. d=days. Est = established. h=hours. ICV=intracerebroventricular. Inf=infusion. IP = intraperitoneal injection. MA = middle age. min = minutes. m=months. MB=methylene blue. NR=not reported. OA = Old Age. PBA=phenylbutyric acid. PO= per oral. SC=subcut. Riva = rivastigmine. Rx = treatment. WT = wild type. YA = young adult.

Supplementary Table 2 – Results of animal studies testing the effect of Macrophage Colony Stimulating Factor (M-CSF) (e), Granulocyte Macrophage Colony Stimulating Factor (GM-CSF) (f), methylene blue (g), geranygeranylacetone (GGA) (h), dantrolene (i), phenylbutyric acid (j) and minocycline (k) on cognition.

	**Author, year**	**Cognitive tests**	**Outcomes**	**Significance**
e	Boisson-neault 2009	T-water maze reversal learning (# trials)	Pre-AD M-CSF 7.9 (0.3), ctrl 14.2 (1.0)	++
			Est AD M-CSF 14 (1.5), ctrl 21 (2.5)	+
		T-water maze escape latency final trial (s)	Pre-AD M-CSF 5.8 (0.5) ctrl 10.5 (0.5)	+++
			Est AD M-CSF 9.5 (1.0), ctrl 14.5 (1.0)	++
		Nesting behaviour (1-5, 5=best)	Pre-AD M-CSF 4.5 (0.1), ctrl 2.8 (0.4)	++
			Est AD M-CSF 3.9 (0.2), ctrl 2.5 (0.2)	++
		Passive avoidance (s)	Pre-AD - NR	NA
			Est AD M-CSF 220(20), ctrl 125(20)	+
f	Boyd 2010	Radial arm water maze (RAWM)		
		Test block 1 Trial 5 (errors)	GM-CSF 2.2 (0.3), ctrl 3.8 (0.6)	+
		Test block 2 Trial 5 (errors)	GM-CSF 1.2 (0.3), ctrl 2.5 (0.2)	+
		Trial 4 overall (errors)	GM-CSF 1.6 (0.4), ctrl 3.25 (0.5)	+
		Trial 5 overall (errors)	GM-CSF 1 (0.2), ctrl 3.2 (0.3)	+
		Cognitive interference task (RAWM interspersed w/ Y-maze)		
		3-trial recall (errors)	GM-CSF 2.1 (0.1), ctrl 3.75 (1)	+
		Proactive interference (errors)	GM-CSF 1.75 (0.5), ctrl 2.5 (1)	±
		Retroactive interference (errors)	GM-CSF 2 (0.5), ctrl 3.3 (0.7)	±
		Delayed recall (errors)	GM-CSF 0.8 (0.3), ctrl 3.5 (0.3)	+
g	Deiana 2008	Morris water maze	(SEMs omitted in original)	
		Path length day 4 (cm)	Ctrl + veh - 600	
			Scop + veh - 1090	
			Scop + MB4 - 550	++
			Scop + MB1 - 620	++
			Scop + MB0.5 - 760	+
			Scop + MB0.25 - 900	±
			Scop + MB0.15 - 890	±
			Ctrl + MB4 - 880	±
			Scop + MB + Riv - 600(50)	+/±
			Ctrl + MB + Riv - 390(20)	±
		Time in target quadrant (%) 1.5hr probe trial	Ctrl + veh - 40(6)	
			Scop + veh - 23(2)	
			Scop + MB4 - 36(5)	+

			Scop + MB1 - 34(2)	+
			Scop + MB0.5 - 30(2)	±
			Scop + MB0.25 - 25(2)	±
			Scop + MB0.15 - 31(2)	±
			Ctrl + MB4 - 35(4)	
			Scop + MB + Riv - 33(1)	++
			Ctrl + MB + Riv - 47(6)	++
g	Medina 2011	Morris water maze		
		Escape latency day 5 (s)	Tg MB 21(2), ctrl 25(3)	±
		Escape latency 24hr probe trial (s)	Tg MB 20(2), ctrl 28(5)	+
		Platform location crosses (#)	Tg MB 4.5(0.5), ctrl 2.5(0.5)	+
		Time spent in target quadrant (s)	Tg MB 22(2), ctrl 17(1)	+
g	Stack 2014	Contextual fear conditioning (% time freezing)	Tg ctrl 32.5(5)	
			Tg MB low 47.5(5)	+
			Tg MB high 27.5(5)	±
g	Hoch-grafe 2015	Morris water maze	TauΔK	
		Path length day 4 training (cm)	Pre1.5m - MB 775(125), ctrl 1100(125)	+
			Pre9m - MB 775(100), ctrl 890(150)	±
			Est15m - MB 1300(50), ctrl 1310(200)	±
			TauRDΔK	
			Pre1.5m - MB 760(60), ctrl 930(180)	±
			Est12m & Est15m	NR
		Probe trial % time in target quadrant	TauRDΔK MB 32(3), ctrl 35(4)	±
h	Hoshino 2013	Morris water maze		
		Escape latency day 7 (s)	GGA 20(5), ctrl 28(4)	±
		Time in quadrant (%)	GGA 39.5(3), ctrl 27.5(2.5)	+
		Times crossing platform (#)	GGA 4.25(0.75), ctrl 3 (0.75)	+
h	Sun 2017	Morris water maze		
		Escape latency day 5 (s)	Tg ctrl - 52(4)	
			Tg+GGA200 - 28(4)	+
			Tg+GGA400 - 26(2)	+
			Tg+GGA800 - 26(3)	+
		Platform crosses (#)	Tg ctrl -0.4(0.1)	
			Tg+GGA200 - 1.4(0.3)	+
			Tg+GGA400 - 2.7(0.7)	+
			Tg+GGA800 - 3.1(0.4)	+
		Object recognition test (% preference)	Tg ctrl - 51(2)	
			Tg+GGA200 - 70(6)	+
			Tg+GGA400 - 69(3)	+

			Tg+GGA800 - 72.5(4)	+
		Y-maze (% alternation)	Tg ctrl - 47.5(3)	
			Tg+GGA200 - 55(5)	±
			Tg+GGA400 - 72.5(7.5)	+
			Tg+GGA800 - 70(10)	+
i	Peng 2012	Morris water maze		
		Escape latency day 5 (s)	Tg dant 5.5(0.5), veh 18(3)	+/±
		Platform crosses (#)	Tg dant 3.5(0.2), veh 1.6(0.5)	+
		Time spent in target quadrant (%)	Tg dant 45(4), veh 31(3)	+
		21 day trial dependent learning procedure (time saved in matching trials (s))	Tg dant short 18(4) long 19(4), veh short 2(6) long 2.5(5)	+/±
i	Hopp 2014	Morris water maze		
		Path length to platform day 4 of training (cm)	YA dant 450(70), veh 250(50)	±
			OA dant 695(40), veh 810(50)	+/±
			YA dant 1.45(0.5), veh 1.4(0.4)	NR
		Time spent at platform location during trial (s)	OA dant 0.4(0.05), veh 0.2(0.05)	NR
			YA dant 27(5), veh 29(3)	NR
		Time spent in annulus around platform (s)	OA dant 15(2), veh 20(2)	NR
i	Wu 2015	Morris water maze	21m (end of treatment)	
		Escape latency day 5 (s)	Tg Dan 19(1), veh 16(2)	±
			WT Dan 27(3), veh 33(5)	±
		Time in target quadrant (%)	Tg Dan 33(3), veh 36(4)	±
			WT Dan 28(3), veh 29(5)	±
			22m (1m post treatment)	
		Escape latency day 5 (s)	Tg Dan 20(3), veh 20(3)	±
			WT Dan 26(3), veh 31(6)	±
		Time in target quadrant (%)	Tg Dan 47(4), veh 40(8)	±
			WT Dan 39(3), veh 39(4)	±
j	Wiley 2011	Hybrid water maze		
		Escape latency day 5 (s)	Wt PBA 11(1), ctrl 17(1)	++
			AD PBA 19(1.5), ctrl 28(1)	+++
k	Choi 2007	Morris water maze		
		Escape latency day 5 (s)	Sham mino 5.5(0.5), ctrl 6(0.5)	±
			AD mino 6(0.5), ctrl 12(0.5)	+
		Time in target quadrant (s)	Sham mino 32.5(2.5), ctrl 34(1.5)	±
			AD mino 34.5(1), ctrl 24.5(2)	+
		Passive avoidance test (s)	Sham mino 235(45), ctrl 230(40)	±
			AD mino 210(30), ctrl 100(40)	+

+++ favouring intervention, highly significant p<0.001. ++ favouring intervention, significant p<0.01. + favouring intervention, significant p<0.05. +/± trend favouring intervention, p<0.1. ± not significant. +/± trend favouring control, p<0.1. - favouring control, significant p<0.05. -- favouring control, significant p<0.01. --- favouring control, highly significant p <0.001. MA = middle age. NA = not applicable. NR = p value not reported. OA = Old Age. YA = young adult.
